# The effects of subjective socioeconomic status and its underestimation on physical activity: evidence from China

**DOI:** 10.3389/fpubh.2026.1850111

**Published:** 2026-06-02

**Authors:** Guo Ziyu, Duan JiaQi, Yang Qian

**Affiliations:** 1 School of Economics and Management, Shanghai University of Sport, Shanghai, China; 2 School of Physical Education, Shandong University, Jinan, Shandong, China

**Keywords:** China, physical activity, social inequalities, subject socioeconomic status, subject status underestimation

## Abstract

**Background:**

Improving levels of physical activity (PA) benefits health and well-being and contribute to attainment of global non-communicable diseases targets and a number of the sustainable development goals. While objective socioeconomic status (OSS) is recognized as a significant factor influencing PA, research on subjective socioeconomic status (SSS) remains limited, particularly regarding the impact of the discrepancy between SSS and OSS (subjective status underestimation, SSU). Consequently, this study investigates the relationship between SSS, SSU and PA in China.

**Methods:**

Using data from 42,442 respondents across five waves of the Chinese General Social Survey (CGSS), we assessed how SSS and SSU are associated with PA and the underlying behavioral and cognitive mechanisms driving this relationship while exploring variations in these effects across age groups.

**Results:**

The findings indicate that SSS was positively associated with PA, whereas SSU showed a negative association with PA. Although the association of SSS was weaker than that of OSS, SSU still diminished the positive relationship of OSS improvement on PA. For behavioral mechanisms, higher SSS and lower SSU were associated with more leisure frequency, and higher SSS further reduced overtime work. Although higher SSU increased part-time work, this indirect effect was not significant. For cognitive mechanisms, SSU inhibits PA by reducing social trust and perceived fairness and by increasing perceived hostility, whereas SSS promotes PA only through reducing perceived hostility. Additionally, the heterogeneity analysis results indicates that while SSS positively correlated with PA across all cohorts, its effect was comparatively stronger among younger adults (ages 18–35). Conversely, SSU was negatively associated with PA in younger groups but exhibited a positive association among older adults.

**Conclusion:**

This study demonstrates how SSS and SSU are associated with PA, highlighting the critical role of subjective perceptions. Enhancing population PA requires interventions targeting both objective conditions and subjective cognitive mechanisms.

## Introduction

Compelling evidence demonstrates that physical inactivity significantly elevates the risk of major non-communicable diseases—including coronary heart disease (6%), type 2 diabetes (7%), breast and colon cancers (10%), and reduces life expectancy (9%) (1). Despite widespread acknowledgment of benefits of physical activity (PA), 31% of the global adult population (1.8 billion people) fail to meet the WHO recommendation of at least 150 min of moderate-intensity activity weekly, representing a 5% increase since 2010 (2). Crucially, inactivity displays a distinct social gradient, with males, higher-educated individuals, and affluent groups maintaining advantages while disadvantaged populations exhibit systematically lower PA levels (3–6. These persistent disparities necessitate moving beyond individual-level explanations to examine structural determinants, particularly how socioeconomic status perpetuates PA inequalities.

Existing research identifies objective socioeconomic status (OSS) as a key factor in PA inequalities (7–10. Despite some debate persists, most studies indicate higher-OSS individuals are generally more physically active than their lower-OSS counterparts, engaging more frequently in both sport and physical activity (3, 11, 12. This disparity can be attributed to the fact that higher-OSS individuals often place greater importance on physical activity participation (13). Conversely, lower-OSS individuals face greater barriers, including health limitations, neighborhood safety concerns, and limited knowledge of health (14, 15), more likely to smoke, to be physically inactive, and to be obese (16, 17). Furthermore, OSS also shapes the PA types: higher-OSS groups favor high-status pursuits like golf (18), while lower-OSS groups gravitate toward less facility-dependent activities such as basketball, soccer, running, and weight training (19).

However, socioeconomic status exerts its profound influence not merely through objective materials but also via subjective identification (20). Subjective socioeconomic status (SSS) refers to an individual’s self-perceived position within the social hierarchy, a core aspect of how he or she thinks of the self and relates to the social world. A growing body of research has shown that SSS predict class-based patterns in self-rated and physiological health even after accounting for OSS (21–24. To complicate matters, although SSS is primarily derived from OSS, the two are not always consistent (25, 26). For instance, only half of Americans accurately identify their OSS, with others either overstating or understating it (27). Furthermore, populations in formerly socialist countries exhibit consistently lower SSS, irrespective of current economic conditions or inequality levels (28).

The discrepancy between SSS and OSS can be understood through the lens of the social cognitive theory of social class (29). This theory posits that the feelings of relative deprivation or advantage are not stemming solely from objective resource disparities, but arise from the subjective construal of one’s rank relative to others. This process hinges on social comparisons—specifically, the individuals to whom they compare their material wealth and how they perceive their own resources in relation to that group (30, 31). Based on the above subjective construal, the misperception between SSS and OSS can be categorized into three types: overestimation, underestimation, and accurate perception. From a social governance perspective, subjective socioeconomic underestimation (SSU) is a consequential phenomenon, as a growing body of research has linked it to detrimental outcomes, including increased hostile and aggressive responding as well as greater negative affect and poorer health (32, 33).

China’s rapid economic development and restructuring have driven significant differentiation in economic benefits and occupational structures, solidifying social stratification as an objective reality. Although SSS-OSS discrepancy is globally common, research specific to China reveals a distinct pattern: compared to the prevalent “middle-class identification” in Western societies, the Chinese populace exhibits a strong tendency to underestimate their economic standing during social comparisons (34). In the meantime, traditional Chinese culture emphasizes the ethos of “diligence and frugality,” which fosters more conservative consumption and savings strategies among individuals (35). People tend to prioritize leisure and entertainment only after securing what they perceive as “sufficient” economic capital. This tendency is particularly pronounced among those who erroneously underestimate their socioeconomic status, leading them to engage in excessive work and savings beyond actual needs—often at the expense of health and leisure pursuits. A survey by the General Administration of Sport of China indicates that as of 2024, only 38.5% of the Chinese population regularly participates in physical exercise, significantly below the global average. Given that the cognitive processes underlying social comparison are known to vary by culture, investigating this phenomenon within China’s distinct socio-cultural backdrop provides an ideal setting to understand the public health implications of status underestimation in non-Western contexts.

Within this context, SSS and SSU likely influences PA through distinct cognitive and behavioral mechanisms. Cognitively, low SSS and high SSU can erode individuals’ fundamental perceptions of social structure, gradually diminishing their trust in societal institutions and sense of fairness, while weakening their feeling of social belonging (36). In China’s cultural environment—which emphasizes social harmony and interpersonal relationships (*guanxi*)—this often leads to withdrawal from community life and social interactions. Such disengagement directly undermines individuals’ self-efficacy and sense of entitlement to participate in community-based or leisure-time physical activities. Behaviorally, this perceived socioeconomic disadvantage may also shape specific lifestyles characterized by compensatory work-leisure time allocation strategies, adopted either to cope with perceived economic instability or to strive for upward mobility. Although economically rational, such behavioral adaptations result in “time poverty,” which directly crowds out opportunities for structured leisure activities such as PA. Consistent with this, a survey conducted by the General Administration of Sport of China identified “lack of time” as the most frequently cited barrier to sports participation.

Despite the clear risks posed by low-SSS and high-SSU, a significant gap persists in the extant literature. While a body of research has sought to identify the independent effect of SSS on PA (37, 38), much of this work operates under a critical limitation: it largely overlook the potential systematic discrepancy between SSS and OSS. Therefore, this study investigates how SSS and SSU are associated with PA in China. We further examine the underlying behavioral and cognitive mechanisms driving this relationship and explore potential variations in these effects across different age groups.

## Methods

### Data

The data for this study were derived from the Chinese General Social Survey (CGSS), a nationally representative longitudinal academic survey conducted by the China Survey and Data Center at Renmin University of China. As the first large-scale, continuous social survey in China, the CGSS collected extensive information on economic conditions, demographic characteristics, health status, and livelihood patterns across 28 provinces, municipalities, and autonomous regions.

We utilized data from five waves of the CGSS (2015, 2017, 2018, 2021, and 2023), mainly for the following reasons. Firstly, as China’s flagship national fitness policy—the “National Fitness Program”—is updated every 5 years, our selected waves span three complete policy cycles. This longitudinal coverage ensures strong representativeness and continuity, enabling accurate tracking of evolving trends in China’s social environment, economic conditions, and residents’ health outcomes. Secondly, the inclusion of pre-pandemic (2015, 2017, 2018) and post-pandemic (2021, 2023) waves allows for systematic control of period effects and major event impacts, particularly regarding COVID-19’s disruption to physical activity patterns (39). This temporal distribution enhances the robustness of findings across different historical contexts. Finally, the 2023 data represent the most current available measurements, capturing the latest developments in socioeconomic and environmental transitions.

After excluding observations with responses coded as “unable to answer,” “refused to answer,” “do not know,” or missing data on core variables, as well as those aged over 90 years, the final sample size used in this study was 42,442. The sample sizes across the five survey waves were 9,353 (2015), 10,940 (2017), 11,071 (2018), 6,122 (2021), and 4,956 (2023)^1^. Although some temporal variation in sample size is evident, this study accounts for potential year-specific heterogeneity by incorporating year-fixed effects in the models. Furthermore, as the analysis does not involve cross-year comparisons, the variation in sample size across waves does not compromise the validity of the findings.

### Measures

#### Explained variables

The explained variable in this study was PA, measured based on self-reported sports participation frequency. This metric was selected as it was the sole consistent measure of PA available across all waves of the CGSS survey from 2015 to 2023, ensuring data comparability. In the CGSS, respondents indicated how often they engaged in sports activities over the past year, selecting from the following categories: “every day,” “several times a week,” “several times a month,” “several times a year or less,” or “never.”

Following the World Health Organization (WHO) guidelines, which recommend that adults engage in at least 150–300 min of moderate-intensity or 75–150 min of vigorous-intensity PA weekly (or an equivalent combination), we classified respondents who exercised “every day” or “several times a week” as meeting the recommended activity level (coded as 1). Those reporting lower frequencies (“several times a month,” “several times a year or less,” or “never”) were categorized as insufficiently active (coded as 0).

#### Explanatory variables

The explanatory variable was SSS and SSU. SSS was measured through the survey item: “Overall, which social stratum do you believe you currently occupy in society?” yielding a continuous variable ranging from 1 (lowest stratum) to 5 (highest stratum), where higher values denoted elevated SSS.

SSU was operationalized as the negative discrepancy between SSS and OSS. Given that the measurement of OSS is intended not to capture OSS itself but to construct SSU with SSS, we selected personal income as the most representative and feasible indicator (40), measured into annual quintiles (1 = lowest 20%, 5 = highest 20%). The raw underestimation value (−4 to +4) was calculated by subtracting OSS (1–5) from SSS (1–5), with negative values designating underestimation. This raw value was then standardized into a continuous 1–5 scale (1 = lowest, 5 = highest), with higher values representing stronger underestimation.

#### Mechanism variables

To examine how SSS and SSU affects sports participation through behavioral and cognitive pathways, we operationalized six mediating variables across two dimensions.

Within the behavioral dimension, individuals with lower SSS and higher SSU often adopt time-based compensation strategies—such as extended work hours or multiple jobholding—to mitigate SSS deficits. These labor supply adjustments systematically erode discretionary time for leisure. We captured this mechanism through three variables: (1) Overtime as a binary indicator assigning 1 to respondents exceeding China’s statutory 44 h weekly work threshold, (2) Part-time work quantifying concurrent paid employment engagements, and (3) Leisure was annual frequency of relaxation activities.

Within the cognitive dimension, individuals with lower SSS and higher SSU typically experience declining social trust, weakened fairness perceptions, and heightened perceived hostility, which constitutes socio-ecological barriers predisposing such individuals to physical inactivity. Accordingly, we capture this mechanism through three variables: (1) Social trust was measured through the survey item: “To what extent do you agree that most people in society can be trusted?”, (2) Perceived fairness was measured through the item: “How fair do you consider contemporary society to be?”, and (3) Perceived hostility was measured through the survey item: “Do you agree that in this society, if you’re not careful, people will find ways to take advantage of you?”. All three variables are continuous variables ranging from 1 to 5, with 5 indicating the highest level and 1 the lowest level.

#### Control variables

The control variables in this study encompass key socioeconomic and demographic factors. Socioeconomic control variables included education, Communist Party of China (CPC) membership, and income—three central indicators of OSS. These variables are closely associated with, yet conceptually distinct from, our core variables of SSS and SSU. Controlling for them helps isolate the effect of objective materials, thereby allowing a clearer identification of the independent influence of subjective perception on PA.

Education, as a core component of human capital, directly affects an individual’s ability to acquire basic skills of PA and understand their long-term health benefits. Income provides the economic foundation for PA, determining one’s capacity to bear related costs. In the Chinese context, CPC membership often entails greater social capital and organizational resources, which may have substantially influence on PA. Therefore, we controlled for education (binary: 1 = completed 9-year compulsory education, 0 = otherwise), CPC membership (1 = member, 0 = otherwise), and income (log-transformed personal annual income to insure the normality and homoscedasticity).

In addition to socioeconomic factors, we included several individual-level demographic characteristics: gender (1 = male, 0 = female), age (continuous, in years), ethnicity (1 = ethnic minority, 0 = Han), marital status (1 = with spouse, 0 = without spouse), and self-rated health (5-point ordinal scale from 1 = poor to 5 = excellent). These factors represent standard controls in social behavioral research (26, 36). Gender and age shape both physiological capacity and preferences for physical activity. Ethnicity may reflect cultural traditions and activity preferences, while marital status influences leisure choices through shifts in household responsibilities and time allocation. Notably, self-rated health is a crucial control variable. It serves both as a precondition for and an outcome of PA, and is also closely associated with SSS and SSU.

### Analytical strategy

All data analyses were conducted using Stata/SE 15.1 software. Descriptive statistics were employed to summarize all the variables in the dataset. For the empirical analysis, we conduct regression model via the Equation 1.


$$Y_{\mathrm{it}}=\alpha_{0}+\alpha_{1}{\mathrm{SSS}}_{\mathrm{it}}+\alpha_{2}{\text{Control}}_{\mathrm{it}}+\sigma +\tau +\varepsilon$$ (1)

In which *α* 0 is a regression constant, $Y_{\mathrm{it}}$ is the indicator for PA, ${\mathrm{SSS}}_{\mathrm{it}}$ represents explanatory variables including SSS and SSU, ${\text{Control}}_{\mathrm{it}}$ is a vector of control variables as depicted above. $\alpha_{1}$ captures the impact of ${\mathrm{SSS}}_{\mathrm{it}}$ . In addition, we controlled for year and region fixed effects to account for the influence of unobserved temporal trends, major events (i.e., the COVID-19 pandemic), and geographic confounders. σ indicates year effects, τ indicates region effects and ε the error term.

## Results

### Descriptive statistics

According to Table 1, only 35.4% of the sample engaged in weekly PA, indicating a relatively low overall participation rate. The mean values for SSS and OSS were 2.429 and 2.985, respectively, yielding an average underestimation gap of 0.556. This represents an 18.6% downward bias in SSS among Chinese residents—a pattern consistent with prior research on class identification underestimation in China (34). Regarding control variables, gender distribution, marriage rates, and educational attainment levels were balanced and aligned with broader demographic trends, supporting the sample’s representativeness.

**Table 1:** The description of variables.

Variable	Mean	Std. dev.	Min	Max
PA	0.354	0.478	0	1
SSS	2.429	0.870	1	5
SSU	3.278	0.753	1	5
OSS	2.985	1.399	1	5
Overtime	0.072	0.258	0	1
Part-time work	0.037	0.359	0	29
Leisure	3.453	0.919	1	5
Social trust	3.524	1.002	1	5
Perceived fairness	3.230	1.026	1	5
Perceived hostility	3.019	1.071	1	5
Gender	0.469	0.499	0	1
Age	51.616	16.690	18	90
Ethnicity	0.925	0.263	0	1
Marriage	0.757	0.429	0	1
Education	0.652	0.476	0	1
Political status	0.115	0.319	0	1
Self-rated health	3.512	1.090	1	5
Income	8.312	3.890	0	13.816

### Empirical results

Table 2 presents the association between SSS, SSU and OSS on PA. Model 1 indicates that SSS is positively associated with PA (*p* < 0.01). Model 2 demonstrates a significant negative relationship between SSU and PA (*p* < 0.05). Model 3 reveals that OSS is positively linked to PA (p < 0.01), with a stronger association than SSS. It can be observed that while improvement in OSS significantly promotes PA, SSU counteracts its positive impact. Additionally, higher PA levels were observed among older individuals, Han Chinese, those with higher education, Communist Party members, individuals reporting better health, and higher-income groups, whereas marriage was associated with reduced PA.

**Table 2:** Effect of SSS, SSU, and OSS on PA.

Variant	(1) PA	(2) PA	(3) PA
SSS	0.173*** (0.013)		
SSU		−0.046** (0.021)	
OSS			0.205*** (0.015)
Gender	−0.001 (0.022)	−0.019 (0.022)	−0.042* (0.022)
Age	0.012*** (0.001)	0.012*** (0.001)	0.014*** (0.001)
Ethnicity	0.241*** (0.043)	0.247*** (0.043)	0.204*** (0.043)
Marriage	−0.246*** (0.025)	−0.236*** (0.025)	−0.248*** (0.025)
Education	0.797*** (0.028)	0.821*** (0.028)	0.743*** (0.028)
Political status	0.556*** (0.033)	0.594*** (0.033)	0.538*** (0.033)
Self-rated health	0.182*** (0.011)	0.205*** (0.011)	0.195*** (0.011)
Income	0.006** (0.003)	0.015*** (0.004)	−0.048*** (0.005)
Year effects	Controlled	Controlled	Controlled
Region effects	Controlled	Controlled	Controlled
*R* ^2^	0.0658	0.0625	0.0657
Observations	42,442	42,442	42,442
Mean VIF	1.27	1.41	1.63

***p < 0.01; **p < 0.05; *p < 0.1; Standard errors are contained within parentheses. Predictor variables, SSS and SSU. Outcome variable, PA.

We further investigated the behavioral and cognitive mechanisms through which SSS and SSU influence PA. As presented in Table 3 regarding behavioral mechanisms, SSS is negatively linked to overtime work (*p* < 0.01), while SSU shows a significant positive relationship with part-time work (*p* < 0.01). These findings indicate that individuals with lower SSS are more likely to engage in overtime-requiring occupations, whereas those have higher SSU undertake more part-time work. Furthermore, we observed that SSS is positively and SSU is negatively linked to leisure time allocation (*p* < 0.01), consistent with empirically observed patterns: groups with higher subjective class status prioritize quality of life, resulting in greater leisure participation.

**Table 3:** Behavioral mechanisms.

Variant	(1) Overtime	(2) Overtime	(3) Part-time work	(4) Part-time work	(5) Leisure	(6) Leisure
SSS	−0.162*** (0.028)		0.002 (0.002)		0.053*** (0.005)	
SSU		0.065 (0.046)		0.015*** (0.003)		−0.040*** (0.009)
Control variables	Controlled	Controlled	Controlled	Controlled	Controlled	Controlled
Year effects	Controlled	Controlled	Controlled	Controlled	Controlled	Controlled
Region effects	Controlled	Controlled	Controlled	Controlled	Controlled	Controlled
*R* ^2^	0.3788	0.3773	0.0098	0.0103	0.0453	0.0435
Observations	42,442	42,442	42,442	42,442	42,442	42,442

***p < 0.01; **p < 0.05; *p < 0.1; Standard errors are contained within parentheses. Predictor variables, SSS and SSU. Outcome variables, overtime, part-time work and leisure.

Table 4 presents the cognitive mechanisms. It is found that SSS is positively related to social trust and perceived fairness but negatively related to perceived hostility; conversely, SSU exhibits the opposite pattern. These findings align with our theoretical propositions that individuals with low SSS and high SSU have a significant social capital disadvantage.

**Table 4:** Cognitive mechanisms.

Variant	(1) Social trust	(2) Social trust	(3) Perceived fairness	(4) Perceived fairness	(5) Perceived hostility	(6) Perceived hostility
SSS	0.092*** (0.006)		0.194*** (0.006)		−0.096*** (0.006)	
SSU		−0.149*** (0.010)		−0.263*** (0.010)		0.119*** (0.010)
Control variables	Controlled	Controlled	Controlled	Controlled	Controlled	Controlled
Year effects	Controlled	Controlled	Controlled	Controlled	Controlled	Controlled
Region effects	Controlled	Controlled	Controlled	Controlled	Controlled	Controlled
*R* ^2^	0.0339	0.0336	0.0645	0.0564	0.0156	0.0132
Observations	42,442	42,442	42,442	42,442	42,442	42,442

***p < 0.01; **p < 0.05; *p < 0.1; Standard errors are contained within parentheses. Predictor variables, SSS and SSU. Outcome variables, social trust, perceived fairness and perceived hostility.

Considering that the stepwise method alone does not provide a formal significance test for the indirect effect, we further conducted bootstrap mediation analyses with 500 resamples and bias-corrected (BC) confidence intervals using the sgmediation command in Stata. For each hypothesized pathway, we computed the indirect effect and its 95% BC confidence interval. A pathway is considered to have a significant mediating effect if the confidence interval does not contain zero. The bootstrap results, including the point estimates of indirect effects and their confidence intervals, are summarized in Table 5. SSS promotes PA by reducing overtime, increasing leisure time, and lowering perceived hostility. SSU inhibits PA by reducing leisure time, social trust, perceived fairness, and increasing perceived hostility. The pathways via part-time work (for SSU) and via social trust and perceived fairness (for SSS) are not supported.

**Table 5:** Bootstrap results.

Mechanisms	Ind. eff.	Std. err.	*z*	BC	CI	Supported
SSS → overtime → PA	0.000**	0.000	1.98	0.000	0.001	Yes
SSU → part-time work → PA	0.000	0.000	1.41	−0.000	0.001	No
SSS → leisure → PA	0.004***	0.000	9.58	0.003	0.005	Yes
SSU → leisure → PA	−0.003***	0.000	−6.26	−0.004	−0.002	Yes
SSS → social trust → PA	0.000*	0.000	1.86	−0.000	0.001	No
SSU → social trust → PA	−0.001***	0.000	−2.94	−0.001	−0.000	Yes
SSS → perceived fairness → PA	0.000	0.000	1.15	−0.000	0.001	No
SSU → perceived fairness → PA	−0.001***	0.000	−3.91	−0.002	−0.001	Yes
SSS → perceived hostility → PA	0.000**	0.000	2.23	0.000	0.001	Yes
SSU → perceived hostility → PA	−0.001***	0.000	−3.33	−0.001	−0.000	Yes

***p < 0.001; **p < 0.05; *p < 0.1.

Table 6 reports the heterogeneity analysis results based on age differences. The findings indicate that SSS consistently promotes sports participation across all age groups, with the strongest effect observed among the 18–35 age group. Conversely, the relationship between SSU and PA exhibits greater age heterogeneity: specifically, SSU enhances sports participation in individuals over 60 years old while inhibiting it among those aged 18–35.

**Table 6:** Heterogeneous impact.

Variant	(1) PA ( 18 ≤ Age ≤ 35 )	(2) PA ( 18 ≤ Age ≤ 35 )	(3) PA ( 35 < Age < 60 )	(4) PA ( 35 < Age < 60 )	(5) PA ( Age ≥ 60 )	(6) PA (Age ≥ 60)
SSS	0.246*** (0.030)		0.164*** (0.019)		0.149*** (0.022)	
SSU		−0.214*** (0.049)		0.023 (0.032)		0.107*** (0.036)
Control variables	Controlled	Controlled	Controlled	Controlled	Controlled	Controlled
Year effects	Controlled	Controlled	Controlled	Controlled	Controlled	Controlled
Region effects	Controlled	Controlled	Controlled	Controlled	Controlled	Controlled
*R* ^2^	0.0649	0.0606	0.0684	0.0654	0.1010	0.0989
Observations	8,731	8,731	19,578	19,578	14,133	14,133

***p < 0.01; **p < 0.05; *p < 0.1; Standard errors are contained within parentheses. Predictor variables, SSS and SSU. Outcome variable, PA.

Table 7 presents robustness checks. Columns (1)–(3) re-estimate the main models using ordinary least squares models. Columns (4) and (5) exclude income from the control set to avoid overlap with OSS: column (4) reports logit estimates, and column (5) reports OLS estimates. Across all specifications, the direction, magnitude, and statistical significance of the coefficients for subjective class and status inconsistency remain stable, confirming that our core findings are not driven by the choice of estimator or by the inclusion of income.

**Table 7:** Robustness test.

Variant	(1) PA	(2) PA	(3) PA	(4) PA	(5) PA
SSS	0.036*** (0.003)				
SSU		−0.008* (0.004)			
OSS			0.044*** (0.003)	0.087*** (0.009)	0.020*** (0.002)
Income	Controlled	Controlled	Controlled	Uncontrolled	Uncontrolled
Other control variables	Controlled	Controlled	Controlled	Controlled	Controlled
Year effects	Controlled	Controlled	Controlled	Controlled	Controlled
Region effects	Controlled	Controlled	Controlled	Controlled	Controlled
*R* ^2^	0.0817	0.0778	0.0818	0.0641	0.0799
Observations	42,442	42,442	42,442	42,442	42,442

***p < 0.01; **p < 0.05; *p < 0.1; Standard errors are contained within parentheses. Predictor variables, SSS, SSU, and OSS. Outcome variable, PA.

## Discussion

While numerous existing studies have emphasized the influence of socioeconomic status on sports participation and acknowledged the importance of SSS, they have not thoroughly examined the mechanisms that SSS influences PA. Furthermore, while no previous research has investigated the impact of the discrepancy between SSS and OSS (SSU). Utilizing data from five waves of the Chinese General Social Survey (CGSS), this study examined the relationship between SSS, SSU and PA, thereby addressing critical gaps in the current research perspective.

To begin, our study found that SSS had a positive correlation with PA. This finding aligns with previous research (37, 38), confirming the significant relationship between SSS and health behaviors. A growing body of research suggests that traditional components of socioeconomic status do not adequately capture underlying factors that determine one’s social position (41). SSS not only takes into account facets of OSS, but also captures multidimensional psychosocial aspects of social status not directly measured by OSS (42, 43). Numerous studies indicate that health behaviors are more closely related to individuals’ subjective identification than material resources (26, 44, 45), which is argued to have socio-psychological effects on health independent from material circumstances.

Continuing, our findings demonstrate that SSU was negatively related to PA. Although no direct literature corroborates this specific relationship, support for its converse proposition exists in prior research. Studies confirm that when SSS exceeds OSS, individuals exhibit increased engagement in healthy behaviors (46). This behavioral adherence may be driven by a tendency to emulate perceived normative conduct of higher-status groups, consequently strengthening behavioral intentions toward PA (47). It is important to acknowledge that while our analysis does not address endogeneity directly, we contend that reverse causality is unlikely to be severe. The rationale is that transient health behaviors, such as PA, are less likely to significantly alter an individual’s fundamental social-psychological positioning within a short time frame. They are thus less plausible as determinants of one’s contemporaneous sense of SSS and SSU. This directionality aligns with the consensus in the literature (37, 38), which typically treats such health behaviors as outcomes rather than causes of these deep-seated social-psychological constructs.

However, our results demonstrate that OSS exhibits greater explanatory power than SSS in accounting for PA. This presents a contrast to the prevailing view. We posit that in the specific context of China, objective structural barriers—such as time constraints, limited economic means, and insufficient access to sports facilities—rather than low subjective identification or participation willingness, may constitute the more fundamental obstacles constraining PA. For instance, residents in China’s smaller cities commonly face more severe constraints regarding sports facilities (48); within such groups where objective conditions are significantly limited, the predictive power of SSS on actual PA behavior is likely relatively weak. Nevertheless, although the influence of SSS is less pronounced than that of OSS, our analysis reveals that SSU still diminishes the positive effect of OSS improvement on PA. This aligns with relative deprivation theory, which posits that underestimation in SSS can significantly undermine the impact of objective improvements.

In addition, we further discovered the behavioral and cognitive mechanisms through which SSS and SSU were associated with PA. For behavioral mechanism, we found that, on the one hand, individuals with lower SSS are more likely to work overtime. This finding aligns with prior research documenting how socioeconomic status shapes occupational preferences—specifically, that affluent individuals prefer occupations that enhance social hierarchies rather than attenuate them (49). On the other hand, those with higher SSU engage in more part-time work. Overtime typically constitutes an involuntary, employer-imposed constraint, where part-time work represents a deliberate strategy to augment economic resources. This compensatory labor pattern aligns with previous studies showing that lower socioeconomic groups tend to sacrifice leisure-time PA for occupational PA (50, 51). However, while the overtime pathway robustly mediates the effect of low SSS on PA, the part-time pathway does not show a significant indirect effect. This null mediation may be explained by the specific nature of part-time work in China, where many part-time jobs (e.g., delivery riders, gig workers) are physically demanding and thus may inadvertently contribute to overall PA, offsetting the time-crowding mechanism. Moreover, the low prevalence of part-time work in our sample (≈2.5%) may limit statistical power.

For cognitive mechanism, SSS was positively related to social trust and perceived fairness but negatively related to perceived hostility, while SSU exhibits the opposite pattern. However, when examining the mediating roles of different types of cognitive mechanism variables, a key asymmetric pattern emerges: while lower levels of social trust and perceived fairness significantly mediate the inhibitory effect of SSU on PA, the higher levels of social trust and perceived fairness associated with SSS do not significantly promote PA. This finding is supported by Andrade et al. (52), who found that a greater perception of social cohesion was associated with increased leisure-time physical activity only among participants of the lowest socioeconomic status. In other words, in the context of physical activity in China, the lack of positive cognitive resources is more explanatory than their abundance. Concretely, for high-SSU individuals, subjective feelings of deprivation and distrust intensify concerns about discrimination and unfair distribution of resources, directly reducing their willingness to engage in public physical activity (53, 54). Conversely, for individuals with high SSS and correspondingly high social trust and perceived fairness, PA depends more on ample time, economic capital, and health awareness, rather than on further increments of trust or perceived fairness. Thus, once these cognitive variables exceed a certain threshold, their marginal promoting effect on PA tends to level off. In contrast, perceived hostility—as a negative cognitive appraisal—mediates both the promoting effect of SSS on PA and the inhibiting effect of SSU on PA. This indicates that the impact of negative cognitive resources does not follow the logic of “lack matters more than abundance”; instead, it exerts a consistent influence across groups with different SSS.

Based on an investigation of age heterogeneity, we found that SSS was positively correlated with PA across all cohorts, with significantly stronger effects observed among younger adults (18–35 years) relative to middle-aged (36–59 years) and older adults (60+ years). This differential impact may be attributable to younger individuals’ less consolidated class identification and stronger endorsement of opportunity beliefs and upward mobility narratives (55), rendering their behavioral patterns more responsive to SSS than OSS. Conversely, SSU exhibited divergent age-specific effects: it significantly inhibited PA participation in younger cohorts while demonstrating a positive association among older adults. For younger adults, PA represents a high-opportunity-cost behavior competing with income-generating activities. SSU amplifies work prioritization through perceived necessity of economic accumulation, thereby suppressing leisure-time PA. Whereas for older people, the positive effect of SSU on PA needs to be understood within the specific Chinese context. Specifically, China’s old-age security system exhibits significant urban–rural disparities and coverage gaps (56). Many older adults face low pension levels, high out-of-pocket medical expenses, and substantial costs for chronic disease management. At the same time, it is very common for older adults in China to take care of their grandchildren, which restricts their daily activity radius to areas near schools and homes (57). This is especially true for older adults with high SSU, who not only experience stronger perceived financial pressure but also have more fragmented disposable time. Therefore, for older people with high SSU, PA becomes a low-threshold, low-cost, and time-fragmented form of activity. It functions as a low-cost health preservation strategy, while also promoting social participation and social recognition, thereby counteracting the negative self-evaluation associated with SSU through a psychological compensation mechanism (58).

## Conclusion

This study reveals how SSS and SSU are associated with PA in China and the underlying behavioral and cognitive mechanisms driving this relationship. Accordingly, public health interventions should systematically incorporate subjective cognitive factors—particularly class perception biases—into intervention designs, with targeted focus on their mediating effects on labor supply decisions and social cognition formation. Concurrently, addressing detrimental impacts of SSU among adolescents requires urgent priority. Implementing corrective initiatives to mitigate distorted self-perceptions could disrupt intergenerational cycles of health disparities.

### Limitations and future research directions

This study has several limitations that warrant acknowledgment. First, PA was assessed using a single-item self-reported frequency measure, which fails to capture intensity, duration, and activity types, and may be subject to social desirability bias. OSS was measured using personal income only, without incorporating education or occupation. While this simplification was necessary for constructing a scalarly comparable SSU variable with SSS, it does not fully capture the multidimensional nature of OSS. Future studies should employ more objective, multi-faceted PA measures and composite OSS indices to replicate and extend our findings.

Second, although this study utilizes multiple waves of CGSS data, the research design cannot fully address endogeneity concerns. To strengthen causal inference, future research could seek to identify valid instrumental variables for status perception discrepancy and apply more rigorous econometric models, such as the IV approach.

Third, while the urban–rural dichotomy remains a defining structural feature of Chinese society, this study could not fully explore its implications. Although regional controls were included, the lack of more granular geographic information limited our ability to conduct a heterogeneity analysis across hukou groups. Future research should focus on this dimension by collecting more targeted samples among various hukou groups.

## Data Availability

Publicly available datasets were analyzed in this study. This data can be found here: https://www.cnsda.org/index.php?r=projects%2Findex&Projects%5Btitle%5D=%E4%B8%AD%E5%9B%BD%E7%BB%BC%E5%90%88%E7%A4%BE%E4%BC%9A%E8%B0%83%E6%9F%A5&Projects%5Bcid%5D=&Projects%5Bcollection_date%5D=&Projects%5Bgeographic_coverage%5D=&bt-search=%E9%AB%98%E7%BA%A7%E6%90%9C%E7%B4%A2.

## Footnotes

1 The small analytic sample sizes for CGSS 2021 and 2023 stem from different issues. In 2021, COVID-19 severely disrupted face-to-face fieldwork, reducing the sample. In 2023, the survey split roughly equally between telephone and face-to-face interviews, many variables were only asked of the face-to-face subsample, leaving them missing for nearly half of all respondents and drastically shrinking the analytic sample after listwise deletion.
